# Naturally gluten-free flours are commonly contaminated, while commercially produced gluten-free flours are relatively safe: a market-based study in Turkey

**DOI:** 10.3389/fnut.2025.1707584

**Published:** 2025-12-11

**Authors:** Hacer Yalçimin Öcal, Hülya Gökmen Özel

**Affiliations:** Faculty of Health Sciences, Hacettepe University, Ankara, Türkiye

**Keywords:** celiac disease, commercially produced gluten-free, gluten contamination, naturally gluten-free, shelf proximity

## Abstract

**Background:**

Gluten contamination and unintentional gluten exposure are serious health concerns for patients with celiac disease. Gluten-free (GF) products may become contaminated at any stage, including harvesting, production, storage, or sale. This study, conducted in Turkey, aimed to compare gluten contamination levels in commercially produced gluten-free (CGF) and naturally gluten-free (NGF) flours—both packaged and unpackaged—across different grain types, and to evaluate the influence of point-of-sale and storage conditions on contamination levels.

**Methods:**

A total of 163 flour products, including oat, buckwheat, corn, and rice flours (*n* = 54 CGF, *n* = 56 unlabeled/packaged NGF, and *n* = 53 unlabeled/unpackaged NGF), were analyzed for gluten content using the EU-approved R5 ELISA method.

**Results:**

Gluten contamination was detected in 16.67% of CGF flours, 50% of unlabeled/packaged natural GF flours, and 84.91% of unlabeled/unpackaged natural GF flours. CGF flours were significantly less contaminated than NGF flours (*p* < 0.001). However, 16.67% of CGF flours exceed the 20 mg/kg threshold, indicating that gluten-free labeling alone does not always guarantee safety. Oat and buckwheat flours had significantly higher contamination levels than corn and rice flours (*p <* 0.001). Although GF flours stored in dedicated sections had lower gluten levels, the difference was not statistically significant. A negative relationship was observed between proximity to gluten-containing products and gluten levels in unpackaged NGF flours (*p <* 0.001). Additionally, gluten contamination increased significantly in unlabeled/unpackaged flours when shared spoons were used (*p =* 0.001).

**Conclusion:**

Gluten contamination is common in NGF products, whereas CGF products are relatively safe. These findings highlight the importance of controlled production and storage practices as well as strengthened verification and certification procedures for gluten-free labeling to minimize the risk of gluten contamination.

## 1 Introduction

Celiac disease (CD) is a chronic immune-mediated enteropathy characterized by gastrointestinal and extraintestinal symptoms, as well as sensitivity to gluten (1, 2). The prevalence of CD, which affects approximately 1% of the general population, has increased in recent years and is now considered a global health concern (3). Consistent with these global trends, a community-based screening study of adults in the Central Anatolia region of Turkey reported that approximately 1% of adults had previously undiagnosed CD (4). Patients with CD are likely to develop hypersensitivity to the toxic effects of gluten and exhibit individual-specific immune responses (5). Studies have reported that in celiac patients, a daily gluten intake of 10 mg is the lowest dose capable of inducing harmful effects on the small intestine mucosa (6), and that gluten intake exceeding 50 mg/day may have toxic effects during treatment (7). Scientific data indicate that foods with gluten levels below 20 mg/kg (ppm) are considered safe for celiac patients. This threshold value is the legal limit used in labeling gluten-free (GF) products and is recognized by the Turkish Food Codex, the Codex Alimentarius (CXS 118–1979), and the European Commission (EU) Regulation no. 828/2014. The production and labeling of GF products in Turkey are regulated and monitored by competent national authorities. In this study, only domestically produced flour samples were purchased, and evaluations were based on the 20 mg/kg limit.

Gluten is a storage protein found naturally in cereals such as wheat, barley, rye, and their hybrids (8). Commercially produced gluten-free (CGF) and naturally gluten-free (NGF) products can become contaminated at any stage of production, processing, transportation, storage, distribution, or sales, and the gluten content may exceed 20 mg/kg (9, 10). Contamination may result from contact with previously contaminated surfaces, from personnel transferring contaminants between zones, or through airborne dust and aerosol particles. In addition, proximity to gluten-containing products, compromised packaging, and airborne gluten dispersions may increase the risk of contamination. Contact with inadequately sanitized pallets and transport equipment, as well as handling multiple products with shared tools and attire, may also contribute to gluten transfer (11).

These various sources of contamination indicate that products marketed as GF may not consistently remain below the 20 mg/kg threshold. This situation limits celiac patients’ access to safe GF products and directly affects the feasibility of maintaining the GF diet in daily life. In Turkey, patients frequently report challenges related to limited access to appropriately labeled GF products at points of sale, limited safe options when eating out or traveling, and feelings of social isolation related to dietary restrictions (12). These access and safety-related barriers highlight the need for further studies examining contamination risks in commonly consumed products, which provides the rationale for this study.

This study extends the scope of previous research on gluten contamination. Earlier studies have reported contamination levels in various food categories collected from different points of sale (13–17). However, in addition to reporting contamination prevalence, the simultaneous evaluation of environmental and operational factors across points of sale is still needed. In Turkey, the use of shared equipment and processing facilities for gluten-containing products, as well as the sale of unpackaged flour without protective packaging, are common. Therefore, this study aims to compare CGF flours with unlabeled/packaged and unlabeled/unpackaged NGF flours using equivalent sample sizes, and to investigate the effects of shelf proximity to gluten-containing products, the storage of GF products in separate sections, and the use of shared spoons for unpackaged products on contamination levels. To our knowledge, no previous study in Turkey has evaluated these conditions together. By addressing these factors, this research highlights risks that individuals with CD may face and underscores the need for preventive strategies and improved sanitation practices in the food industry and at points of sale.

## 2 Materials and methods

### 2.1 Collection of flour samples

Samples of oat, buckwheat, corn, and rice flours, which are commonly used in GF diets and produced domestically, were purchased from various points of sale, including supermarkets, local grocery stores, herbalist shops and small grain mills with retail counters. The distance (cm) between GF flours and gluten-containing products at each location, as well as information on the use of shared spoons, was recorded. A total of 163 products were analyzed for gluten content: 54 labeled/packaged commercially GF samples (oat, buckwheat, maize, and rice flours), 56 unlabeled/packaged NGF samples, and 53 unlabeled/unpackaged NGF samples. In this study, the term “labeled” refers to products bearing a GF claim on their packaging, while “unlabeled” refers to products without any GF statement on their label or packaging. Products labeled as gluten-free (commercially produced gluten-free) had the words “gluten-free” and the official gluten-free certification logo (e.g., the Crossed Grain symbol) on them. “Packaged” NGF products are those products that are produced and sold in sealed protective retail packaging, while “Unpackaged” products are flours sold in large open bags or containers and purchased by weight in specific amounts. All flour samples purchased from various points of sale were stored at 4 °C until analysis.

### 2.2 Determination of the gluten content

#### 2.2.1 Sample extraction

The flours were weighed and placed in pre-labeled Falcon tubes. Each sample was assigned a unique code and recorded in Microsoft Excel. For extraction, 2.5 mL of the extraction cocktail solution (Ridascreen R-7006, composed of detergents and reducing agent) was added to 0.25 g of flour. An additional 0.25 g of skimmed milk powder was added to buckwheat and oat flours to account for their tannin and polyphenol contents. The tubes were then vortexed and incubated in a water bath at 50 °C for 40 min.

After cooling to room temperature, 7.5 mL of 80% ethanol was added to each tube. The tubes were mixed for 1 h, then transferred to 1.5 mL Eppendorf tubes, and centrifuged at 2500 × g for 10 min. The resulting supernatants were then transferred to new 1.5-mL Eppendorf tubes and stored at room temperature. Each flour sample was analyzed in duplicate. To minimize the risk of contamination, each batch was processed in separate rooms at different time intervals.

#### 2.2.2 Measurement of the gluten content

After extraction, the samples were diluted to 1.0:12.5 (v/v) with diluent solution. Standards and samples were then loaded into the wells of ELISA plates. After the enzyme had been added to each well, the microplates were washed with the wash buffer. The wells were incubated at room temperature for 30 min, after which substrate and chromogen were added. The reaction was stopped with a stop solution, and absorbance was measured using a microplate reader.

The lowest concentration detectable by the kit was 2.5 ppm (mg/kg) gliadin, equivalent to 5 ppm (mg/kg) gluten. In our data, values <5 ppm and >80 ppm were included, with <5 ppm treated as 2.5 ppm and >80 ppm as 80 ppm, following the methods recommended by the US EPA and CORESTA. Flour samples with gluten values >20 ppm were considered contaminated. Results were calculated according to the manufacturer’s instructions and recorded in an Excel spreadsheet.

The gliadin embedded in the gluten structure of the purchased samples was measured using the Ridascreen Gliadin sandwich R5 ELISA method, validated by the Association of Official Analytical Chemists and the American Association of Cereal Chemists International. Ridascreen Gliadin (art. no. R7001) ELISA kits were used according to the manufacturer’s protocol to determine the gluten content.

### 2.3 Statistical analyses

The data obtained in this study are presented as ratios, means, standard deviations, medians, and ranges. Continuous variables are presented as means ± standard deviations, while skewed or non-normally distributed variables are presented as medians with ranges. Ratios are reported for categorical or proportional data. The Kruskal–Wallis test was used to examine differences in gluten amounts among the flour groups. Dunn’s test was applied for *post hoc* pairwise comparisons when significant differences were found. The chi-square test was used to compare the distribution of contaminated and uncontaminated samples within each group. In addition, Spearman correlation coefficient was applied to assess relationships between numerical variables, such as distance and gluten levels. Statistical analyses were performed using IBM SPSS Statistics for Windows, version 23.0 (IBM Corp., Armonk, N.Y., United States), and statistical significance was set at *p <* 0.05.

## 3 Results

### 3.1 Prevalence of gluten contamination

Gluten levels above the 20 ppm threshold were detected in 50.31% of all flour samples (Table 1). By product classification, 16.67% of CGF flours, 50% of unlabeled/packaged NGF flours, and 84.91% of unlabeled/unpackaged NGF flours contained gluten above 20 ppm (Table 1). Across all flour types, labeled/packaged CGF flours showed significantly lower contamination than NGF flours (*p* < 0.001). By flour type, gluten contamination above 20% was present in 82.9% of oat flour, 59.5% of buckwheat flour, 28.9% of corn flour, and 28.6% of rice flour samples. No CGF rice flour samples showed gluten contamination. Overall, oat flour had the highest contamination frequency, while corn flour had the lowest (Table 1).

**Table 1:** Gluten contamination percentage in commercially produced and naturally gluten-free flour types (%).

Flour types	Gluten contamination (>20 ppm)							
	Labeled/packaged CGF		Unlabeled/packaged NGF		Unlabeled/unpackaged NGF		Total	
	(*n* = 54)		(*n* = 56)		(*n* = 53)		(*n* = 163)	
	c/n	%	c/n	%	c/n	%	c/n	%
All samples	9/54	16.7	28/56	50.0	45/53	84.9	82/163	50.3
Oat	6/13	46.2	14/14	100.0	14/14	100	34/41	82.9
Buckwheat	2/16	12.5	10/13	76.9	13/13	100	25/42	59.5
Corn	1/10	10.0	1/15	6.7	9/13	69.2	11/38	28.9
Rice	0/15	0	3/14	21.4	9/13	69.2	12/42	28.6

c/n = contaminated samples/total samples. CGF: Commercially produced gluten-free, NGF: Naturally gluten-free.

### 3.2 Comparison of gluten contamination among flour types

**Figure 1:**
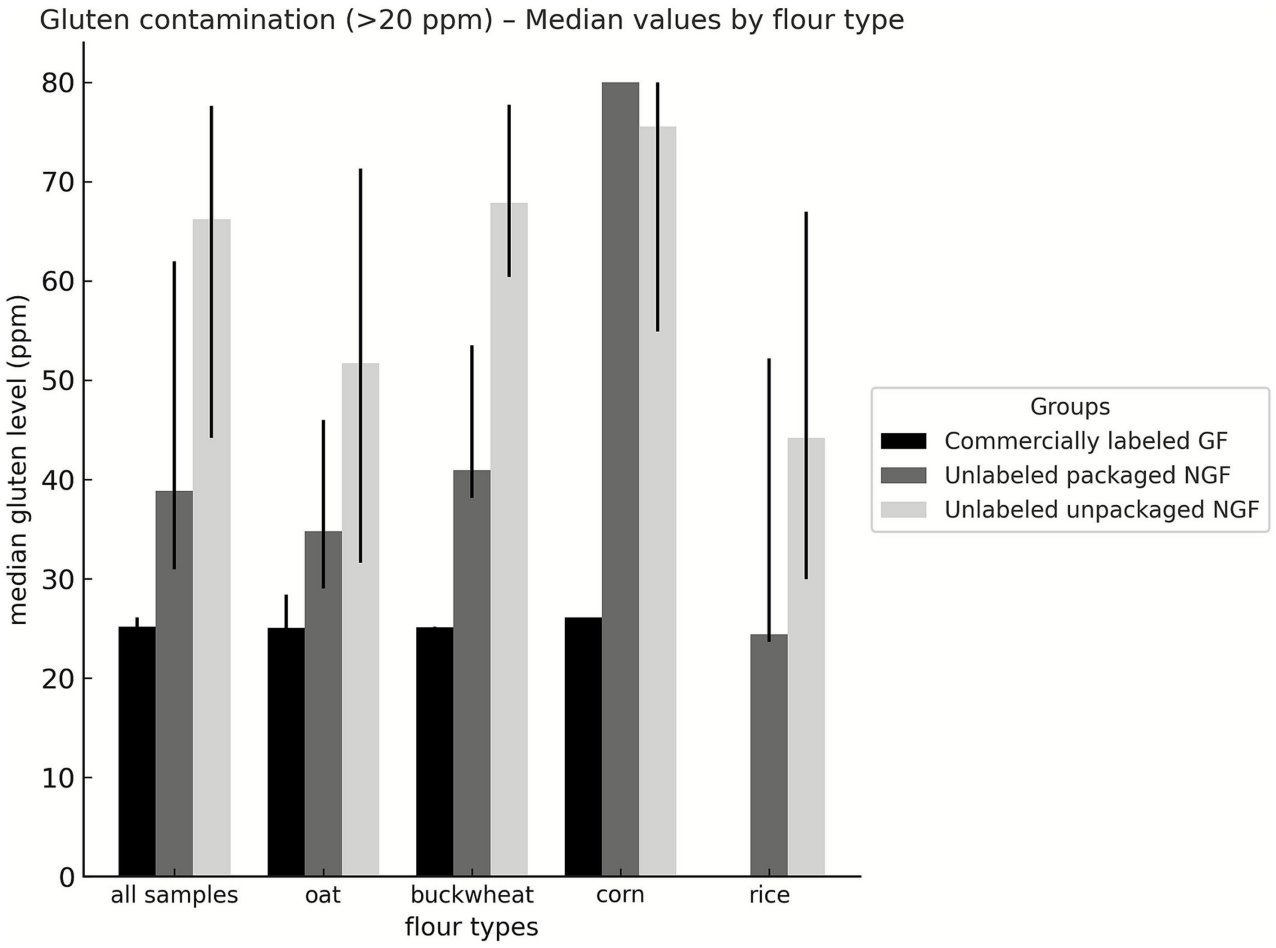
Gluten levels in contaminated commercially produced gluten-free and naturally gluten-free flours (ppm). CGF: Commercially produced gluten-free; NGF: Naturally gluten-free.

Oat flour was significantly more contaminated with gluten than corn and rice flours (Figure 1; *p* < 0.001). Buckwheat flour also had higher gluten contamination than corn (*p* = 0.029) and rice flours (*p* = 0.001). Gluten levels in contaminated samples of both CGF and NGF flours are provided in Supplementary Table 1.

### 3.3 Relationship between gluten levels and shelf proximity

**Figure 2:**
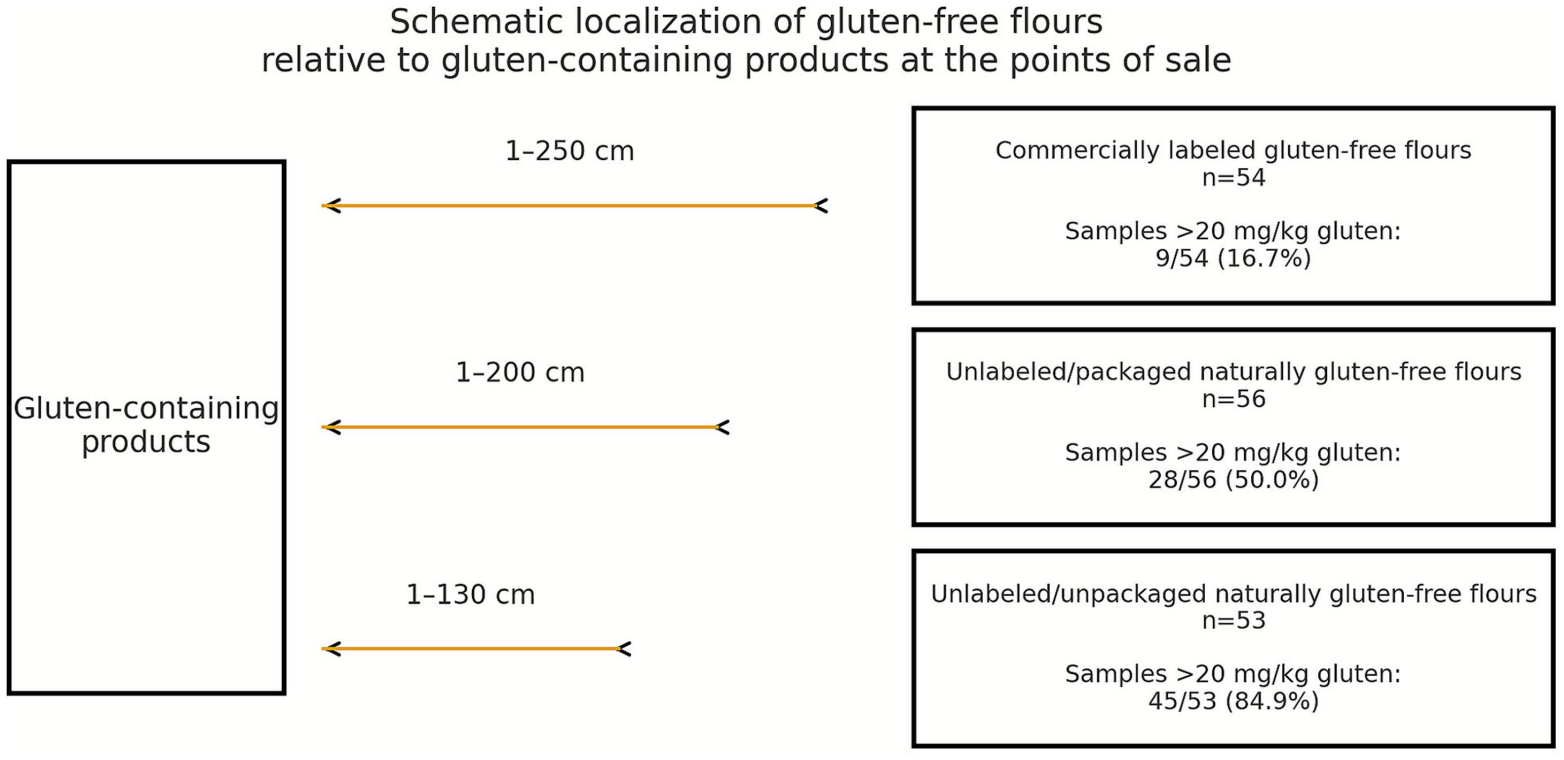
Schematic localization of gluten-free flours relative to gluten-containing products at the points of sale.

The relationship between gluten levels and the proximity of shelves to gluten-containing products was analyzed. No statistically significant association was observed for labeled/packaged CGF flours (Table 2; *p* = 0.088). The corresponding scatter plots are presented in Supplementary Figure 1. Similarly, no significant relationship was found for unlabeled/packaged NGF flours (*p* = 0.171). However, a significant negative correlation was identified for unlabeled/unpackaged NGF flours (*p* < 0.001). A schematic representation of the placement of gluten-free flours in relation to gluten-containing products at the points of sale is presented in Figure 2.

**Table 2:** Relationship between gluten content and shelf proximity to gluten-containing products.

Groups	Gluten content median (Q1-Q3, ppm)	Shelf proximity median (Q1-Q3, cm)	Spearman’s ρ	*p-*value
Labeled/packaged commercially gluten-free (*n* = 54)	2.50 (2.50–13.72)	200 (54–200)	−0.234	0.088
Unlabeled/packaged naturally gluten-free (*n* = 56)	20.91 (2.50–38.85)	11 (1.0–18.5)	0.186	0.171
Unlabeled/unpackaged naturally gluten-free (*n* = 53)	54.42 (29.38–76.80)	25 (7.0–57)	−0.695*	0.001

*Correlation is significant at the *p* < 0.05 level. CGF: Commercially produced gluten-free, NGF: Naturally gluten-free.

### 3.4 Effect of storage in dedicated gluten-free sections

Products were examined based on whether they were stored in a dedicated GF section, and 64.8% of CGF flours were found to be the only flours stored in such sections. As shown in Table 3, gluten contamination levels were lower in products stored in dedicated sections; however, no significant difference in contamination levels was observed between the two groups.

**Table 3:** Comparison of gluten levels according to storage in a dedicated section and the use of shared spoons.

Condition	Gluten amount (ppm)			
	*n* (%)	Median	Q1–Q3	*p-*value*
Dedicated section				
Present	35 (64.8%)	2.50	2.50–7.45	
Absent	19 (35.2%)	12.19	2.50–21.02	*p* = 0.112
Shared spoon use				
Present	40 (75.5%)	67.42	46.04–78.63	
Absent	13 (24.5%)	7.68	2.50–27.89	*p* = 0.001

*Mann Whitney U test.

### 3.5 Effect of shared spoon use

A shared spoon was used in 75.47% of unlabeled/unpackaged NGF flour samples. The gluten content of unpackaged samples without a shared spoon was compared with that of samples with a shared spoon. The use of a shared spoon was found to significantly increase gluten contamination (Table 3; *p* = 0.001).

## 4 Discussion

### 4.1 Prevalence and sources of gluten contamination

Gluten contamination and unintentional gluten exposure are serious health concerns for patients with CD (18). Among food groups, those carrying the greatest risk of gluten contamination are grains, flours derived from them, and processed or cooked grain-based products, as reported in several studies (10, 19). In this study, we examined gluten contamination levels in flour samples from oat, buckwheat, corn, and rice. Overall, 50.31% of flour samples had a gluten content above 20 ppm. This proportion is substantially higher than that (9.5%) reported in a similar study conducted in Canada on flour and starch samples (20). According to a meta-analysis of 40 articles, the prevalence of gluten contamination in GF labeled foods was 9.5% with a 95% confidence interval (10). Several studies on gluten contamination, including this one, have employed the R5 Méndez method. Taken together, these findings indicate that gluten contamination in flour samples marketed in Turkey is more common than reported in Canadian studies and in the global meta-analysis of GF labeled foods.

CGF flours, which are preferred because they are considered safe for use by individuals with CD, were found to have a gluten contamination rate of 16.7%. Similarly, another study conducted in Turkey reported a contamination rate of 17.5% in CGF products (19). The similarity between these two Turkish studies suggests that gluten contamination in CGFs is not incidental but reflects a recurring issue in the local market. International data support this trend; for example, in China (17), 13.4% of foods labeled “gluten-free” exceeded the 20 mg/kg gluten limit, while in Northwest Mexico (16), 17.4% of CGF foods exceeded this threshold. Collectively, these findings indicate that contamination rates in CGF flours in Turkey fall within a similar range to those reported in other countries. For products with gluten levels of 20 mg/kg or more, gluten exposure during production or packaging means that the manufacturers’ GF claims do not reliably ensure compliance with the threshold. The presence of the GF label and logo indicates the manufacturer’s self-declared compliance with the threshold value of <20 mg/kg. However, it remains unclear which analytical methods manufacturers use and how frequently they perform gluten testing. In the present study, all analyses were conducted in an independent laboratory using the Codex Alimentarius–recommended R5 ELISA method. Taken together, these results suggest that the 16.7% rate of contamination observed in GF-labeled products may arise during early stages, such as production and packaging, before products reach the point of sale. This is likely related to the use of shared production lines and equipment. The detection of gluten at levels ≥20 mg/kg in some GF-labeled flours indicates that current verification and certification procedures for gluten-free labeling may be insufficient and need to be strengthened.

Consistent with the existing literature, CGF flours (oat, buckwheat, corn, and rice) were significantly less contaminated than NGF flours (*p* < 0.001). A meta-analysis of 40 articles confirmed that gluten contamination is higher in NGF products than in CGF products (10). This difference is likely due to the use of more controlled production lines separating gluten-containing cereals and sanitation protocols designed to prevent cross-contamination during production.

Previous reports from different regions have also revealed considerable gluten contamination in NGF flours. A study conducted in Southern India (21) found that 35.9% of NGF flour samples were contaminated, while in Canada (20), only 16% of NGF starch and flour samples were contaminated. By contrast, our study found that 50% of unlabelled/unpackaged NGF flours contained gluten levels above 20 ppm, indicating a higher contamination rate than those reported in Southern India and Canada. This potential contamination may occur during transportation and packaging, as these products may pass through a shared production chain with gluten-containing items. Additionally unlabeled/packaged NGF flours were found to be significantly less contaminated than unlabeled/unpackaged NGF flours (*p* < 0.001). In our sample, 84.9% of unlabeled/unpackaged NGF flours were found to be contaminated. These flours may be exposed to flour dust via airborne transfer and shared spoon usage due to the lack of protective packaging.

Among all groups, CGF flours were the most reliable product, consistent with previous reports (9). It is therefore important to select CGF products that remain within the safe daily gluten intake limit of 10 mg, according to Codex Alimentarius standards for patients with CD, especially in populations with high cereal consumption.

### 4.2 Flour-type specific findings

Oats, which are nutritionally valuable cereals rich in soluble dietary fiber, vitamins, and minerals, are a viable option for diversifying cereal consumption in individuals with CD (22). A study conducted in Canada on 133 oat samples, both labeled and unlabeled as gluten-free, reported that nearly 88% contained gluten above 20 ppm (23). In Chile, 36% of products labeled as gluten-free and 66.7% of unlabeled oats had gluten levels exceeding the 20 ppm limit (24). In the United States, the measurable gluten content (<5 ppm) in oats labeled as gluten-free was 11% in 2011, but this rate rose to 35% in 2022. This situation has been attributed to variability caused by drought and decreased oat yields during this period, as well as an increase in invasive weeds that may contain gluten (25). In this study, 82.9% of oat flours exceeded the 20 ppm threshold and were largely responsible for contamination in NGF flours. Based on data obtained from Canada, Chile, the United States, and Turkey, prevalence rates varies across countries; however, oat products are prone to gluten contamination in different regions of the world. Among the oat flours analyzed, the lowest contamination levels were observed in CGF flours. It is therefore advisable for consumers to select packaged products labeled GF to ensure adherence to a GF diet.

A comprehensive evaluation of both *in vivo* and *in vitro* experiments has reported that buckwheat extract may have cytotoxic potential against tumorigenesis, reduce inflammation, promote alpha diversity in the gastrointestinal microbiota, and increase the formation of short-chain fatty acids in stool samples (26). This study demonstrated that 59.5% of purchased buckwheat flour contained gluten above 20 ppm. CGF corn flour exhibited a lower contamination rate of 10% compared with the other flour types. All CGF rice flour samples contained gluten levels below 20 ppm. In another study performed in Turkey, 57% of buckwheat-based products labeled as GF contained gluten above 20 ppm, while 5% of corn-based and 4% of rice-based products exceeded this threshold (19). Similar findings were observed in China; for example, one study from China reported that 50% of buckwheat-based samples were contaminated, whereas rice-based (5%) and corn-based (20%) samples showed lower contamination (17). Consistent with our findings, buckwheat-based products showed higher contamination rates than corn- or rice-based products (17, 19). A possible explanation for the higher susceptibility of oat and buckwheat to contamination could be that they are grown in crop rotations with wheat, rye, and barley, and gluten-containing grains may mix with GF grains during harvest. Furthermore, similarities in processing steps (27) owing to analogous grain structures and the use of the same type of milling equipment (with adjusted settings), and the potential sharing of transportation equipment, production lines, and storage areas also increase the risk of cross-contamination.

A study in Turkey examined the food consumption pattern of patients with CD and found that they consumed 1.6 g/day of buckwheat (28). Daily gluten exposure from the most contaminated flour group, unlabeled/unpackaged GF buckwheat flour, was 0.11 mg. With a consumption of 100 g/day, the estimated exposure would be 6.87 mg of gluten. Considering daily consumption, gluten exposure remained below the safe upper limit of 10 mg/day set by the Codex Alimentarius for patients with CD.

### 4.3 Influence of environmental and operational factors

According to the Turkish Food Codex Regulation on Food Labeling and Consumer Information, gluten must be explicitly declared in labels as a food allergen. Codex Alimentarius standards (CXS 118, CXC80) recommend protective measures such as keeping products containing allergens separate, but no provision requires a dedicated section for products containing gluten or for GF products at points of sale. In this study, labeled GF flours were examined based on whether they were stored in a dedicated GF section; 64.8% of CGF flours were stored in a dedicated GF section. Gluten contamination levels were lower for products stored in dedicated sections; however, no significant difference in contamination levels was observed between the two groups. The level of cross-contamination may be minimized due to the protective packaging of CGF products. However, the shelf life of these products remains unknown. Further studies should investigate the storage duration of GF products located in the same shelves or in the same section as gluten-containing products.

We also examined the effect of shelf proximity to gluten-containing products on the gluten contamination levels of GF flours sold at points of sale. Notably, no significant relationship was observed between shelf proximity and gluten contamination in CGF flours or in naturally unlabeled/packaged GF flours. However, gluten contamination decreased significantly as the distance from gluten-containing products increased in unlabeled/unpackaged NGF flours (*p* < 0.001). Shared spoons were used for unlabeled/unpackaged flours, and because of the insufficient number of samples without shared-spoon usage, a significant cut-off value could not be established. The greater contamination of unlabelled/unpackaged NGF flours is likely to be driven by a combination of mechanisms including increased exposure to airborne flour dust and aerosolized particles when the distance to gluten-containing products decreases, and the use of a shared spoon. A study highlighting the importance of increased exposure to airborne flour dust and aerosolised particles as a potential means of gluten contact reported that individuals living in close proximity to flour mills had higher levels of CD-specific antibodies and experienced CD-related symptoms more frequently (29). Taken together, these findings suggest that inhaling or ingesting airborne flour particles may increase the risk of developing celiac disease.

In a study conducted by Miller et al. in the United Kingdom, gluten contamination increased when GF food was prepared simultaneously in environments where wheat flour was used, with contamination rising as the distance to the wheat flour decreased. The study also recommended a minimum distance of 2 m and the use of barriers to create separate areas for the safe preparation of GF food in commercial kitchens (30).

In our study, the use of a shared spoon with gluten-containing products significantly increased gluten contamination in unlabeled/unpackaged NGF flours (*p <* 0.05). In a study by Studerus et al., kitchens were divided into ‘gluten-containing’ and ‘gluten-free’ areas, separated by 4 m. Across scenarios involving shared knives, colanders, ladles, and wooden spoons, measured gluten concentrations remained below 20 ppm (31). However, when larger quantities of pasta and GF pasta are cooked simultaneously, cross-contamination risk should be considered to increase. Another study indicated that baking gluten-containing and GF pizzas simultaneously or alternately in the same oven does not pose a significant cross-contamination risk (32). Nevertheless, it has been reported that wheat flour residue can become aerosolized when handled, leading to contamination of GF pizza dough and other GF ingredients at multiple production stages. Experimental studies show that gluten transfer is possible through contaminated equipment; however, when proper sanitation was applied, sharing utensils did not result in gluten levels exceeding the 20-ppm threshold (31, 33). Intervention studies from the United Kingdom, Switzerland and Italy indicate that gluten transfer can be kept below 20 ppm under controlled conditions, provided that clear spatial separation and appropriate hygiene practices are ensured. By comparison, our research conducted in retail environments in Turkey shows that such preventive measures are not consistently applied. In addition to airborne gluten transfer in unpackaged flours without shared spoon use, gluten can also be transferred via the spoon surface, potentially due to inadequate cleaning between uses. Furthermore, training is not mandated or provided to personnel at local points of sale.

### 4.4 Limitations and future directions

Our study had some limitations. First, although the sample size was based on statistical power calculations and equitably allocated across groups, research involving broader sampling across multiple geographical regions is needed, as factors affecting gluten contamination may vary regionally. For example, the high contamination levels detected in buckwheat and oat flour may be related to crop rotation practices involving gluten-containing cereals. Given this potential source of variability, regional and temporal differences should not be overgeneralized. While possible factors contributing to contamination at points of sale were evaluated, potential contamination occurring before products reached the point of sale was not addressed.

Additionally, shelf proximity to gluten-containing products and the duration of exposure to these environments may influence gluten contamination, particularly in unpackaged products (30). Another limitation is the lack of data on how long products were kept on the shelf at points of sale. Therefore, future experimental studies should consider both exposure time and proximity.

### 4.5 Strengths and implications for food safety

Despite these limitations, this study has several strengths. These include the collection of adequate samples of various flour types from multiple independent points of sale. In addition, the evaluation of gluten contamination in GF products considers not only prevalence but also sales and storage conditions. Furthermore, the comprehensive assessment of environmental factors, such as shelf proximity to gluten-containing products, storage in dedicated sections, and use of shared equipment, provides novel data that support the development of effective food safety strategies across food production and sales environments.

## 5 Conclusions and recommendations

Owing to inadequate supervision of production processes, co-processing on shared production lines, use of shared equipment, airborne gluten transfer, and insufficient sanitation practices, 50.31% of the flour samples marketed in Turkey had gluten levels above the 20-ppm threshold. Therefore, there is an urgent need to establish regulations and implement appropriate protocols to ensure safe consumption. The situation may differ across countries depending on national regulations and industrial practices. Implementing a systematic monitoring program for GF foods may help improve the quality of life and clinical outcomes of individuals with CD and other gluten-related disorders. It is recommended that manufacturers ensure products remain GF throughout production, transportation, storage, and sale through regular sampling and testing. Although commercially produced GF flours are generally less contaminated than naturally GF flours, independent analyses using the R5 ELISA method revealed that 16.7% of the flours labeled as gluten-free exceeded the 20 mg/kg threshold. Therefore, gluten-free labeling alone does not always guarantee safety, highlighting the need to strengthen procedures for verifying and certifying ‘gluten-free’ claims, particularly during milling, production and packaging.

## Data Availability

The original contributions presented in the study are included in the article/Supplementary material, further inquiries can be directed to the corresponding author.
