# Supplementary material for: Naturally gluten-free flours are commonly contaminated, while commercially produced gluten-free flours are relatively safe: a market-based study in Turkey
Source: Front Nutr. 2025 Dec 11;12:1707584. doi: 10.3389/fnut.2025.1707584 (PMC12738360; doi:10.3389/fnut.2025.1707584)
Supplement: Supplementary file 1 [file Table_1.DOCX]

Supplementary Material

**Supplementary Table 1.** Gluten amount in contaminated commercially produced gluten-free and naturally gluten-free flour types (ppm)

|  | **Gluten contamination (>20 ppm)** | | | | | | | |
| --- | --- | --- | --- | --- | --- | --- | --- | --- |
| **Flour types** | **Labeled/packaged CGF** | | **Unlabeled/packaged NGF** | | **Unlabeled/ unpackaged NGF** | | **Total** | |
|  | **(n=54)** | | **(n=56)** | | **(n=53)** | | **(n=163)** | |
|  | **Median** | **Q1-Q3** | **Median** | **Q1-Q3** | **Median** | **Q1-Q3** | **Median** | **Q1-Q3** |
| **All samples** | 25.20 | 24.82–26.08 | 38.85 | 30.38–67.36 | 66.19 | 44.20–77.65 | 47.49 | 29.49–76.58 |
| **Oat** | 25.04 | 24.17–29.49 | 34.80 | 28.94–47.54 | 51.70 | 31.17–72.03 | 33.44^a^ | 27.89–69.19 |
| **Buckwheat** | 25.11 | 25.02–25.20 | 40,92 | 37.99–56.60 | 67.86 | 60.39–77.72 | 56.60^a^ | 38.56–76.38 |
| **Corn** | 26.08 | 26.08–26.08 | 80.00 | 80.00–80.00 | 75.56 | 54.88–80.00 | 75.56^b^ | 48.83–80.00 |
| **Rice** | - | - | 24.41 | 22.88–80.00 | 44.20 | 29.93–66.97 | 43.70^c^ | 26.89–72.31 |

*Different letters indicate significant differences (p < 0.05).*

*CGF: Commercially produced gluten-free, NGF: Naturally gluten-free.*

**Supplemetary Figure 1.** Relationship between gluten amount and shelf proximity to gluten-containing products


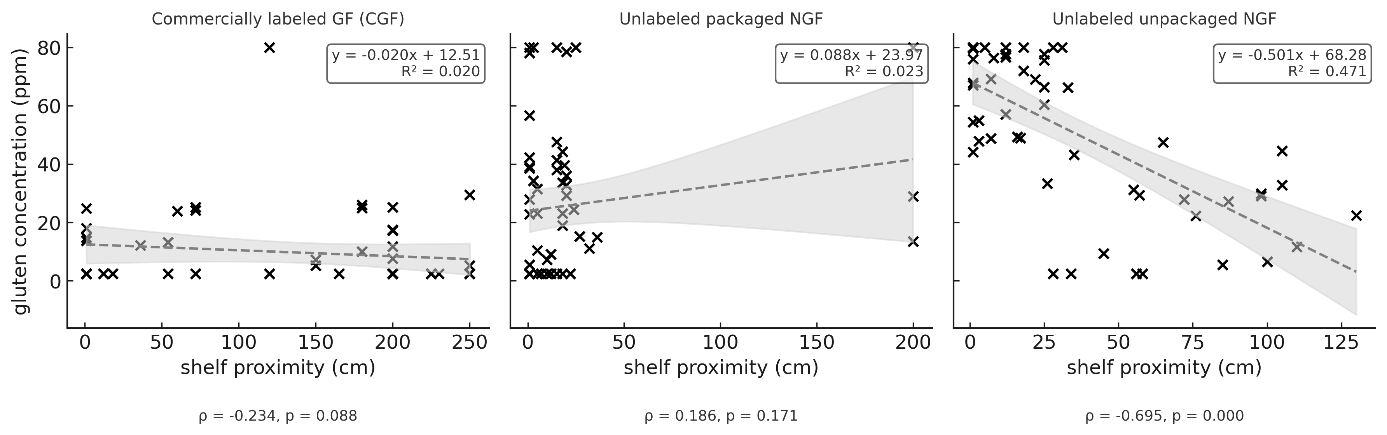


R², indicates the coefficient of determination (proportion of variance in the dependent variable explained by the independent variable). Each panel shows Spearman’s correlation p-value and the regression-derived p-value.
